# Performance evaluation of facile synthesized CA-PVA-GO composite for the mitigation of Cr(Ⅲ) and C.I. acid violet 54 dye from tannery wastewater

**DOI:** 10.1016/j.scenv.2024.100092

**Published:** 2024-06

**Authors:** Fatema-Tuj-Zohra, Monira Akter Swarna, Emamul Mobin

**Affiliations:** Institute of Leather Engineering and Technology, University of Dhaka, Dhaka 1209, Bangladesh

**Keywords:** Adsorbent, CA-PVA-GO composite, Chrome tanning, Dye, Wastewater

## Abstract

Untreated tannery wastewater contains a large amount of toxic metals, dyes, and other pollutants, which pose adverse effects on the ecosystem and public health. In this work, a calcium alginate-poly vinyl alcohol-graphene oxide (CA-PVA-GO) composite was prepared to remove metals and dyes, particularly Cr(Ⅲ) and CI acid violet 54 (AV54) dye, from tannery wastewater. FESEM, FTIR, and XRD analyses were applied to characterize the GO and CA-PVA-GO. Different operational variables, viz. pH (3.0–5.5 for Cr(III) and 2–7 for dye), dosage (0.164–2.46 g/L), contact time (10–60 min), initial concentration (39, 65, 98, and 201 ppm for Cr(III) and 21.5, 38.5, 54.5, and 61.75 ppm for dye), and temperature (298, 308, 318, and 328 K) were studied to evaluate the efficiency of the CA-PVA-GO composite. The optimum conditions for Cr(Ⅲ) and AV54 dye adsorption were found to be pH (5.0 and 3.0), dosage (0.82 g/L for both), and time (45 and 60 min), respectively, with 35.35 ± 1.43% and 84.63 ± 2.54% removal efficiency. The experimental data was analyzed through the Langmuir and Freundlich isotherms. The maximum adsorption capacity (q_m_) was observed at 173.01 and 74.68 mg/g for Cr(Ⅲ) and AV54 dye, respectively. The pseudo-second-order kinetic model was fitted better (R^2^ = 0.981, 0.995, 0.92, and 0.995) than first-order for AV54 dye adsorption. Thermodynamic analyses revealed that the Cr(Ⅲ) and AV54 dye adsorption processes were spontaneous and exothermic. The value of Gibbs free energy (ΔG) for Cr(III) adsorption was obtained at −7.433, −4.508, −2.626, and −1.311 kJ/mol, whereas it was −5.178, −4.867, −4.628, and −4.555 kJ/mol for dye. The values of ΔH and ΔS were −67.257 and −0.198 kJ/mol for Cr(III) and −10.852 and −0.019 kJ/mol for the dye removal. The regenerated CA-PVA-GO composite was reused successfully. Different physicochemical parameters, viz., concentration, pH, TDS, EC, BOD_5_, and COD of chrome tanning and dyeing effluents, were analyzed before and after the adsorption. The results of chromium and dye removal from tannery wastewater were 53.18% and 93.91%, revealing that the developed eco-friendly CA-PVA-GO composite could be an operative adsorbent for tannery wastewater treatment and possibly scaled up to an industrial level.

## Graphical Abstract

**Figure:**
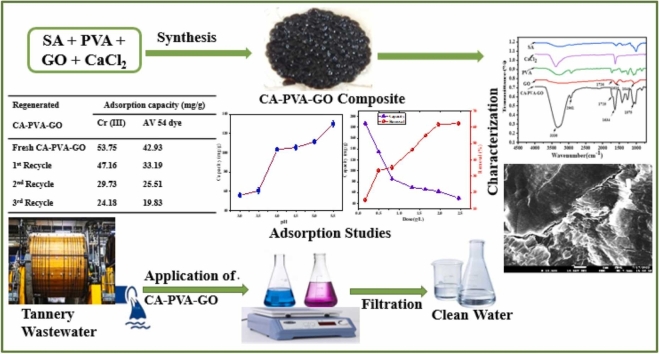

## 1 Introduction

The tanning industry faces significant environmental challenges due to the discharge of pollutants such as chromium and dyes in wastewater. In leather processing, putrescible raw hides and skins are converted to non-putrescible leather through a tanning process that protects it from microbial degradation [1], moisture, heat, and other environmental effects [2]. In leather processing, chrome tanning is the most widespread tanning method. About 80–90% of tanning used basic chromium sulfate (BCS), which created cross-links with collagen fibres and possessed high mechanical properties, dyeing properties, breathability, and better hydrothermal stability [3], [4], [5]. During chrome tanning, only 55–70% chromium [6] is attached to the pickle pelt, and the rest is discharged with wastewater, which contains approximately 2000–5000 mg/L of chromium [7]. Currently, almost 1.6 million tons of 100,000 types of dyes [8] are consumed annually, out of which 10–15% are discharged into water bodies during use [9]. Dye-contaminated post-tanning wastewater discharges a huge amount of suspended solids and biochemical and chemical oxygen demand, which decreases the dissolved oxygen of surface water and also restricts sunlight penetration, reduces photosynthesis, and increases the turbidity of water [10], [11]. The existence of dyes in wastewater enhances the possibility of bioaccumulation, which contaminates aquatic ecosystems and endangers the environment as a whole [12]. Heavy metals, in some cases, cause genuine issues to mankind and the environment. Cr, Pb, Hg, Cu, Cd, Zn, Co, Ni, etc. are generally utilized heavy metals [13], and discharged unexploited heavy metals cause severe risk to the environment [14], [15]. In particular, owing to its higher water solubility and mobility, hexavalent chromium is remarkably carcinogenic than trivalent chromium, although Cr (III) can cause numerous health hazards such as skin allergy, inflammation [16], nausea and metabolic malfunction due to extended exposure [17]. Therefore, the removal of pollutants is highly important for saving the ecosystem and mankind. It is also crucial to save the water on this earth for future generations as well as for the whole network.

The adsorption method is used instead of other techniques such as reverse osmosis, coagulation [18], membrane filtration [19], ion exchange [20], [21], chemical precipitation [22], flotation, and flocculation [23], to eliminate heavy metals and dyes from the wastewater. Adsorption is used due to its reasonable cost, accessibility, conformability in design, high effectiveness, adsorption capacity, and ability to remove a wide range of chemicals [24], [25], [26], [27], [28]. Employments of graphene and graphene-based adsorbents are expanding day by day due to their high adsorption capacity and performances. Graphene-based adsorbents such as graphene oxide (GO) [26], reduced GO [29], [30], GO composites [31], and GO hydrogel [32] are considered promising materials for adsorption. To address the environmental challenges, this research aims to develop an eco-friendly adsorbent, the Calcium Alginate-Poly Vinyl Alcohol-Graphene Oxide (CA-PVA-GO) composite, and investigate its effectiveness in removing pollutants, particularly chromium and acid dye (acid violet 54), from tanning and dyeing wastewater with a focus on preparation methods, adsorbent kinetics, adsorption mechanisms, and regeneration of used adsorbents. Experimental variables like pH, adsorbent dosage, adsorbate concentration, contact time, and temperature were observed to evaluate the adsorption equilibrium process, kinetics, and thermodynamics. The objectives of the study are as follows:

• To prepare an eco-friendly CA-PVA-GO composite adsorbent.
• To investigate the viability of applying the developed CA-PVA-GO composite adsorbent for treating tanning and dyeing wastewater.
• To evaluate the adsorption efficiency of the CA-PVA-GO composite for the removal of chromium and acid violet 54 dye from tannery wastewater.

## 2 Experimental

### 2.1 Materials

Graphite powder (99.5%), hydrazine hydrate (88%), hydrogen peroxide (30%), potassium permanganate, calcium chloride, and polyvinyl alcohol were collected from Merck, Germany; H_2_SO_4_ (98%) and HNO_3_ (65%) from Active Fine Chemicals, Bangladesh; sodium nitrated from Uni-chem, China; HCl (37%) from RCI Labscan, Thailand; and sodium alginate from Research-Lab, Fine Chem. Industries were purchased for the synthesis of CA-PVA-GO composite. Chromium (III) sulfate (Cr_2_(SO_4_)_3_.6 H_2_O), from Qualikems, India, was obtained for making the stock solution. Leather dye C.I. acid violet 54 (AV54) was attained from a local tannery of Tannery Industrial Estate Dhaka (TIED), Savar, Dhaka, Bangladesh. All chemicals were analytical grade and used without any modification.

### 2.2 Methods

#### 2.2.1 Aqueous solution preparation

1000 ppm chromium (III) sulfate standard solution and 100 ppm AV54 dye solution was prepared from Cr_2_(SO_4_)_3_.6 H_2_O and C.I. Acid violet 54 dye using deionized water for experimental analysis.

#### 2.2.2 Synthesis of GO and CA-PVA-GO composite

**Fig. 1:**
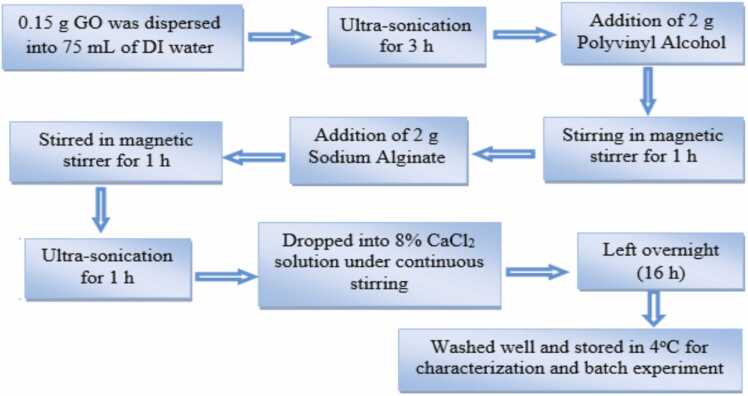
Flow diagram of CA-PVA-GO composite beads synthesis from GO.

**Fig. 2:**
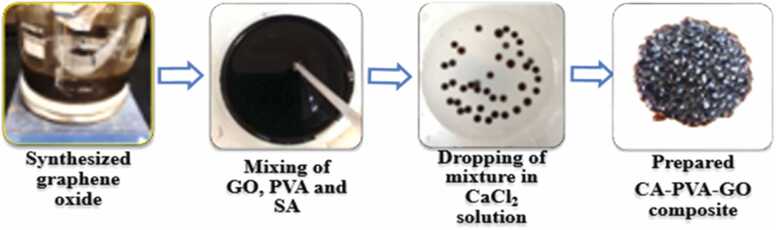
Schematic diagram of CA-PVA-GO composite beads synthesis from GO.

GO was synthesized using a modified Hummer’s method, which was previously reported by the author [26]. Prepared GO (0.15 g) was dissolved into deionized water (75 mL) and treated in the ultra-sonication bath for 3 h to prepare uniform dispersion in the aqueous medium. PVA (2 g) was mixed with the solution, and then sodium alginate (2 g) was added and stirred continuously for 1 h under magnetic stirring. To form a homogeneous dispersion, the mixture was then treated with ultrasonication for 1 hour. Then the homogeneous mixture of CA-PVA-GO was dripped into an 8% CaCl_2_ solution to create beads, which were then cross-linked by a gelation/CaCl_2_-hardening process under uniform stirring to avoid agglomeration. Complete cross-linking was achieved by soaking the beads in CaCl_2_ solution overnight (16 h) [33], [34]. The CA-PVA-GO beads were then collected and washed several times with deionized water before being stored at 40°C for characterization and batch experiments. Fig. 1 and Fig. 2 show the flow diagram and schematic diagram of the synthesis process of CA-PVA-GO.

#### 2.2.3 Cross-linking mechanism of CA-PVA-GO composite

**Fig. 3:**
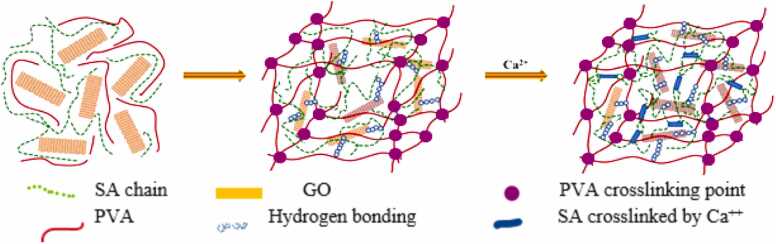
Schematic cross-linking mechanism of CA-PVA-GO [35].

The cross-linking mechanism of the CA-PVA-GO composite has been demonstrated schematically in Fig. 3. Sodium alginate, polyvinyl acetate, graphene oxide, and calcium ion (Ca^++^) have been interconnected and crosslinked through hydrogen bonding and formed the CA-PVA-GO composite beads.

#### 2.2.4 Characterization

##### 2.2.4.1 Fourier transformed infrared (FT-IR) analysis

FTIR spectra of native GO, SA, PVA, CaCl_2_, and CA-PVA-GO composites were recorded using an FTIR spectrophotometer (BRUNKER ALPHA II) in the range 400–4000 cm^−1^ to recognize the functional groups belonging to it.

##### 2.2.4.2 X-ray diffraction (XRD) analysis

XRD of GO and CA-PVA-GO composite was performed by multipurpose X-ray diffraction method (Ultima IV) with Cu Kα radiation in the range 5–70° to signify the crystallinity of GO and composite with 40 kV voltage and 40 mA intensity, scan speed of 10.00°, and scan axis of 2θ.

##### 2.2.4.3 Field-emission scanning electron microscopy (FE-SEM) analysis

A field emission scanning electron microscope (FESEM; JSM-7610 F, JEOL) was utilized for assessing the surface morphology of the CA-PVA-GO composite.

##### 2.2.4.4 Batch adsorption studies

The performance of CA-PVA-GO composite beads was evaluated with batch experiments to determine the optimum pH, dosage, time, concentration, and temperature for optimum adsorption of Cr(Ⅲ) and AV54 dye. In this study, the pH range was 3–6, and the initial dosage of the composite was 1.64 g/L to carry out the process of Cr (III) adsorption by the CA-PVA-GO composite. Moreover, the optimum dosage was evaluated by verifying the dosage from 0.164 to 2.46 g/L. To estimate the optimum time, the adsorption process was carried out for 10–90 min at different concentrations (39.5, 65.25, 98.2, and 201.75 ppm) and temperatures (298, 308, 318, and 328 K) for chromium (III) adsorption. In the case of dye adsorption, the same dosages, time, and temperature were followed except pH (2, 3, 4, 5, 6, and 7) and initial dye concentration (21.5, 38.5, 54.5, and 61.75 ppm). To adjust pH, 0.1 M HCl and 0.1 M NaOH were used. Every experiment was performed at a speed of 150 rpm by an orbital shaker. The filtration process was carried out on Whatman filter paper, and then the filtrate was analyzed. After optimization of the CA-PVA-GO composite, chrome tanning and dye wastewater were studied separately with the CA-PVA-GO composite. Inductively coupled plasma-mass spectrometry (ICP-MS-7900, Agilent Technologies International Japan Ltd., model no. G8403A) was used to analyze chrome tanning filtrate for total chromium concentration. The dye wastewater filtrate was investigated through a UV–visible spectrophotometer (VARIAN-Cary 50). To compute adsorption capacity, the following (1), (2) were used:

(1) $$q=\frac{\left(C_{i}-C_{e}\right)}{W}\times V$$

(2) $$\%\;\;\mathrm{of}\;\mathrm{removal}=\frac{\left(C_{i}-C_{e}\right)}{C_{i}}\times 100\%$$

Here, q = adsorption capacity represented as mg/g. C_i_ and C_e_ = initial and final concentration of chromium / dye in solution (mg/L), V = volume of the solution (L), and W = weight of the adsorbent (g).

## 3 Results and discussion

GO is a single-thickness sheet with a high specific area and a huge number of functional groups attached to the surface. It is an sp2 hybridized two-dimensional element that shows high performance as it has improved electrical, mechanical, hydrological, and thermal characteristics [36] and can eliminate heavy metals (such as Cr, Pb, Cu, etc.) and dyes from wastewater. GO is used as a stuffing that is spread by PVA to form a homogeneous composite by the cross-linking of calcium chloride and also acts as a filler that works as a reinforcing element [37]. Polyvinyl alcohol, as a low-cost, water-soluble, biodegradable, non-toxic, and environment-friendly polymer, is used to incorporate sodium alginate, which is also a biodegradable organic material [38]. Sodium alginate is a water-soluble direct polysaccharide comprised of α-L-guluronate and β-D-mannuronate with hydrophilic, biocompatibility, and nontoxic properties [39]. Creating a natural graphene oxide composite with sodium alginate, polyvinyl alcohol, and calcium chloride could be a good environmental solution for the removal of heavy metals and dyes.

### 3.1 Characterization of CA-PVA-GO

#### 3.1.1 FTIR analysis

**Fig. 4:**
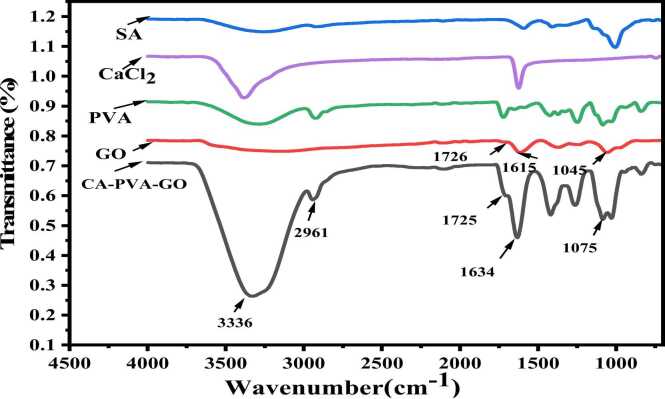
FTIR of CA-PVA-GO composite, GO, PVA, CaCl_2_, and SA.

In Fig. 4 of the FTIR spectra, the main functional groups showed various identical peaks at different wavenumbers, e.g., at 1235 cm^−1^ indicating the epoxy group (-O-stretching), at 1604 cm^−1^ C=C due to aromatic stretching band, at 1737 cm^−1^ C=O because of the carboxylic group, at 3371 cm^−1^ a broad peak of the –OH group was observed, which ensured the oxidation of graphite powder converting to graphene oxide [40], [41]. In the case of SA, at 3319 cm^−1^ and 1528 cm^−1^, the peaks were observed due to the functional groups of -OH, and COO-, respectively. These peaks were stronger in PVA as they held more –OH groups. In the CA-PVA-GO composite, a peak at 2961 cm^−1^ was observed owing to the –CH_2_ group, which enhanced vibrational intensity [42], the band at 1075 cm^−1^ was signifying C-O stretching, 1634 cm^−1^ was presenting C=C group and owing to C=O, a peak was formed at 1725 cm,^−1^ which indicated the presence of GO in composite.

**Fig. 5:**
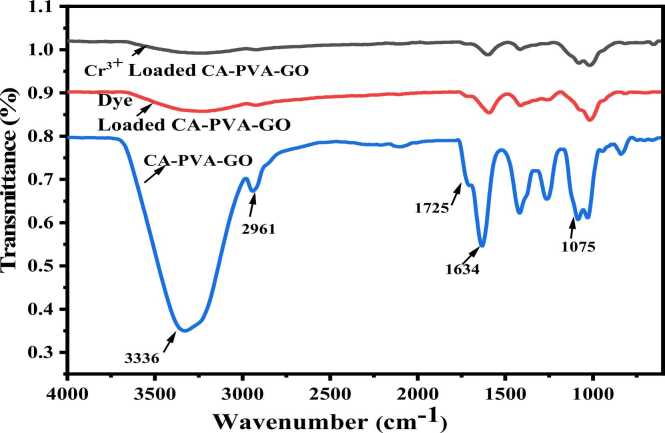
FTIR of CA-PVA-GO composite before and after adsorption of Cr(Ⅲ) and dye.

All identical peaks of the individual components were present in the composite [35]. It was noticed from Fig. 5. that some functional groups moved to a different frequency level or disappeared after Cr (III) and dye adsorption, indicating the potential involvement of the groups in the uptake of adsorbate [43].

#### 3.1.2 FESEM analysis

**Fig. 6:**
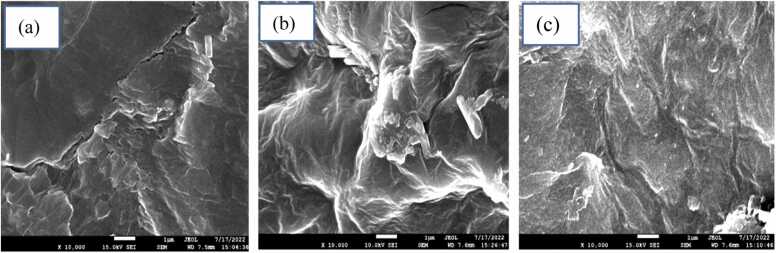
FESEM images of CA-PVA-GO (a) before adsorption (b) after Cr(Ⅲ) adsorption and (c) after dye adsorption.

The surface morphological structure of the CA-PVA-GO composite before and after adsorption of Cr(Ⅲ) and dye was explored through FESEM. Fig. 6 shows the composite images at 10000x magnification. The working distance was 7.5 mm in high vacuum mode with 15.0 kV. Fig. 6(a) shows a denser, porous, and cracky rough structure because of crosslinking GO with alginate and PVA, which facilitated the adsorption process [44]. It was perceived in Fig. 6. (b) and (c) that after Cr (III) and dye adsorption, the surface of the adsorbent was concealed by a layer of materials that could be due to adsorption of metal and dye, respectively [31], [45].

#### 3.1.3 XRD analysis

In XRD, the crystallinity of the composite and the difference between the layers were determined. The oxidation, crystallinity, and complete exfoliation of GO were assessed using XRD.

**Fig. 7:**
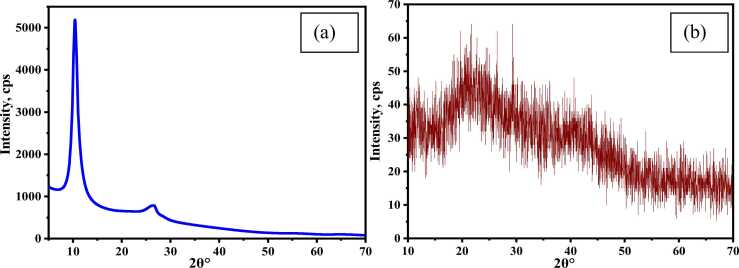
XRD of (a) GO and (b) CA-PVA-GO composite.

The XRD of GO is shown in Fig. 7(a), where a sharp peak is observed at 2θ (10.10^0^) conforming to an interlayer spacing of 8.75 Ȧ, which is consistent with the literature value of 2θ (11.4^0^) and interlayer spacing (7.8 Ȧ) [24], [46]. No sharp peak is formed in CA-PVA-GO, which represents the non-crystallinity of the composite. The wide range of 2θ = 15–25° and a peak at 2θ = 21.74° are perceived in Fig. 7(b), which indicates the diffraction peak corresponds to PVA [47].

### 3.2 Adsorption capacity of CA-PVA-GO composite for Cr (III) and AV54 dye

#### 3.2.1 Effect of pH

**Fig. 8:**
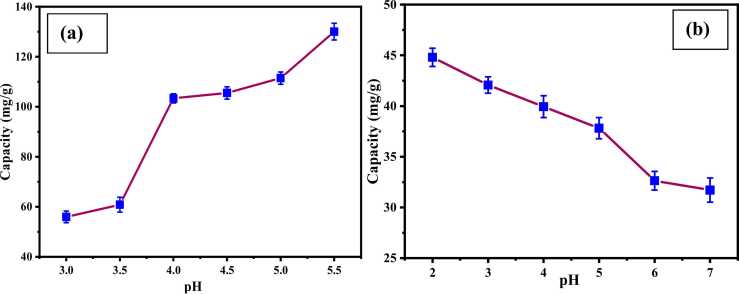
Effect of pH for the removal of (a) Cr(Ⅲ) and (b) AV54 dye.

The impact of pH was studied through the batch adsorption process by changing pH from 3.0 to 5.5 at a 227 ppm initial concentration of Cr_2_(SO_4_)_3**.**_6 H_2_O solution. In the case of dye adsorption, the pH range was 2–7 with an initial concentration of 40.25 ppm, and the CA-PVA-GO composite dosage was 1.64 g/L. A solution’s pH is important because it influences the electrostatic interaction between adsorbent and adsorbate, which affects the adsorption efficiency [48], [49]. Fig. 8(a) demonstrates that the adsorption capacity was increased from 55.94 ± 2.35–111.43 ± 2.49 mg/g with increasing pH from 3.0 to 5.0, and the maximum adsorption capacity was found at 130.03 ± 3.39 mg/g at pH 5.5. The adsorption capacity was observed to be less at a lower pH (3.0) due to the presence of a higher number of protons; moreover, a highly acidic pH is harmful to the aquatic environment and human beings [42]. The presence of a lesser amount of protons at pH 4.0–5.0 leads to increase the adsorption capacity of composite and thus increase the uptake of cationic metal ions due to strong electrostatic attraction. Finally, the optimum pH for Cr(Ⅲ) adsorption was decided to be 5.0, as higher than this pH causes precipitation of chromium and reduction of concentration [50].

Fig. 8. (b) shows the removal efficiency of dye, which was increased at lower pH, as anionic dye molecules could be easily arrested by the positive charge of the adsorbent. The maximum adsorption capacity was observed at 44.81 ± 0.9 mg/g at pH 2.0, while the capacity was decreased gradually with increasing pH and reached 31.71 ± 1.19 mg/g at pH 7.0. At high pH, electrostatic repulsion was generated in the negative charges of the adsorbent, and a solution of anionic dye caused less adsorption [51], [52]. Since the excessive acidic condition is harmful to the environment, the optimum pH for the deduction of dye was decided to be 3.0 as the adsorption capacity (42.07 mg/g) was close to extreme adsorption.

#### 3.2.2 Effects of dosage on adsorption capacity and percentage of removal

**Fig. 9:**
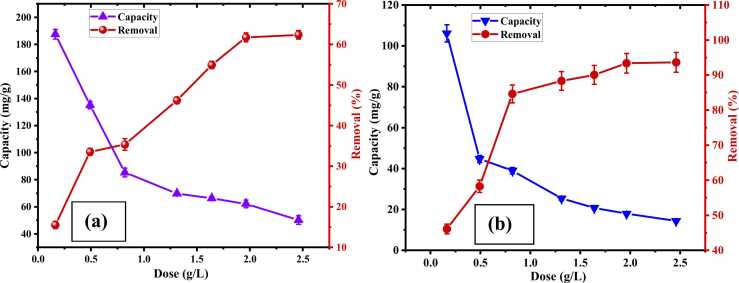
Effect of CA-PVA-GO dosage on adsorption capacity and % removal of (a) Cr (III) and (b) AV 54 dye.

Effects of adsorbent dosage on Cr (III) adsorption were analyzed with different dosages from 0.164 to 2.46 g/L to investigate the optimum dosage. Batch experiments were conducted for 2 hours with an initial concentration of 198 ppm at an optimum pH of 5.0. The % of removal was increased from 15.53 ± 0.9% to 62.37 ± 1.03%, and the adsorption capacity was decreased from 187.50 ± 3.5–50.20 ± 3.1 mg/g with the increased dosage of CA-PVA-GO from 0.164 to 2.46 g/L (Fig. 9. (a)). The optimum dosage was observed at 0.82 g/L with 85.37 ± 3.2 mg/g of capacity. On the other hand, the AV 54 dye removal study was conducted at pH 3.0 with 37.75 ppm concentration and the same dosage of CA-PVA-GO. Dye removal increased from 46.09 ± 1.38% to 93.64 ± 2.81%, while adsorption capacity declined from 106.10 ± 4.24–14.37 ± 0.57 mg/g with increased dosage. Fig. 9. (b) relates that the optimum dosage for dye removal was 0.492 g/L with 44.72 ± 1.78 mg/g of capacity and 58.28 ± 1.75% removal. However, a dosage of 0.82 g/L was continued over the study as dye removal sharply increased to 84.63 ± 2.54% with 38.96 ± 1.56 mg/g adsorption capacity. The availability of additional adsorption sites and greater surface area enhance the removal of Cr (III) and AV 54 dye; however, more unsaturated sites of adsorbent and shortage of adsorbate in the solution lessen the adsorption with higher adsorbent dosage [53].

#### 3.2.3 Effect of time and concentration

The impact of varying concentrations on Cr(Ⅲ) adsorption in solution was investigated using four different concentrations of 39, 65, 98, and 201 ppm. To investigate the optimum Cr (III) concentration, a 0.82 g/L dosage was added to a series of 20 mL standard solutions of Cr (III) of 39, 65, 98, and 201 ppm in identified conical flasks at pH 5.0. In the case of AV54 dye adsorption, 0.82 g/L dosage was added to 20 mL of different concentrations (21.5, 38.5, 54.5, and 61.75 ppm) at pH 3.0. The experiments were accomplished over several durations ranging from 10 to 60 minutes with constant agitation at 150 rpm.

**Fig. 10:**
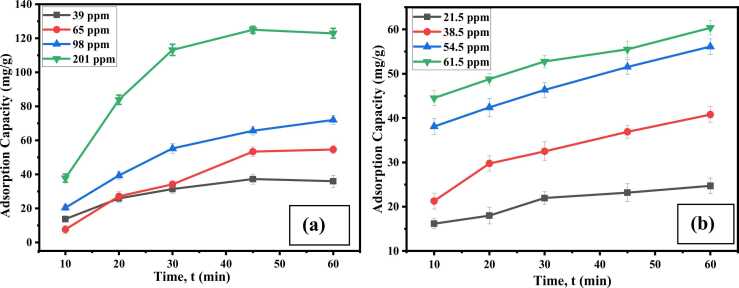
Effect of concentration and contact time for (a) Cr (III) and (b) AV 54 dye removal.

The findings (Fig. 10. (a) and (b)) revealed that adsorption capacity increased with increasing duration until it reached equilibrium. Adsorption capacity was seen to achieve equilibrium immediately after 45 minutes and to increase with increasing concentration. At a lower concentration of 39 ppm, the equilibrium adsorption capacity of Cr (III) was 37.20 ± 2.92 mg/g, while at 201 ppm, the capacity was 125.00 ± 2.01 mg/g. The extreme adsorption capacity for AV54 dye was achieved at 60.37 ± 1.62 mg/g in a 61.5 ppm solution. The adsorption capacity of CA-PVA-GO was increased at higher concentrations due to the presence of enormous adsorbate in the solution, which accumulated available active sites for the adsorbent [54], [55]. Initially, there were more active sites available; however, after a certain time, these sites got saturated [56].

### 3.3 Isotherms studies

To recognize the adsorbate distribution on the adsorbent surface at equilibrium, experimental data was analyzed through Langmuir and Freundlich isotherm models. In Langmuir isotherm, the adsorbate was assumed to be absorbed by the adsorbent in a monolayer well-defined with no intermolecular interactions; however, Freundlich isotherm inferred multilayer adsorption and denoted non-uniform distribution [57], [58].

#### 3.3.1 Langmuir isotherm

In this model, the highest adsorption capacity q_m_ (mg/g) was computed by plotting C_e_/q_e_ against C_e_. Langmuir isotherm is stated in linear form by the Eq. (3).

(3) $$\frac{C_{e}}{q_{e}}=\frac{1}{q_{m}b}+\frac{1}{q_{m}}C_{e}$$

Here, C_e_ = equilibrium concentration of adsorbate (mg/L); q_e_= adsorption capacity at equilibrium (mg/g); q_m_= maximum capacity (mg/g); b is the Langmuir constant. From the Langmuir equation, slope and intercept plot of C_e_ versus C_e_/q_e_ is used_._in calculation of b and q_m_.

Adsorption efficiency and usability of the Langmuir type adsorption process is predicted by the dimensionless equilibrium parameter R_L_, which is defined by the following Eq. (4).

(4) $$R_{L}=\frac{1}{1+C_{m}b}$$

R_L_ defines whether the process is favorable or not. The R_L_ values must be in the range of 0 and 1 for favorable adsorption, while unfavorable, linear, and irreversible adsorption processes indicated by R_L_>1, R_L_=1 and R_L_>0, respectively [59], [60].

**Fig. 11:**
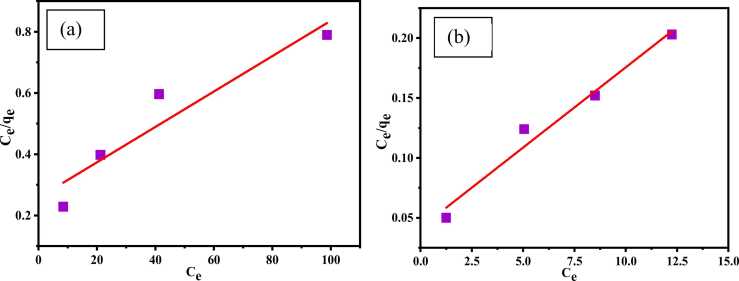
Langmuir isotherm for (a) Cr (III) and (b) AV54 dye removal by CA-PVA-GO composite.

A linear connection between C_e_/q_e_ and C_e_ was observed in Fig. 11 (a) and (b) with an acceptable regression factor; R^2^ was 0.898 for Cr(Ⅲ) and 0.975 for dye. The maximum theoretical adsorption capacity of adsorbent per unit mass (q_m_) was 173.01 and 74.68 mg/g for Cr (III) and AV54 dye, respectively. Separation factor R_L_ was calculated by Eq. (4) and the values were 0.182 and 0.999 for Cr(Ⅲ) and dye, respectively, which signify favorable monolayer adsorption process [61], [62].

#### 3.3.2 Freundlich isotherm

This isotherm assumes multilayer adsorption with heterogeneous distribution, and the adsorption amount depends on the equilibrium concentration of the adsorbate in the solution [49]. This isotherm relates the uptake capacity to the equilibrium concentration commonly used. The Eq. (5) is linearized as follows:

(5) $$\ln q_{e}=\ln k_{F}+\frac{1}{n}\ln C_{e}$$

**Fig. 12:**
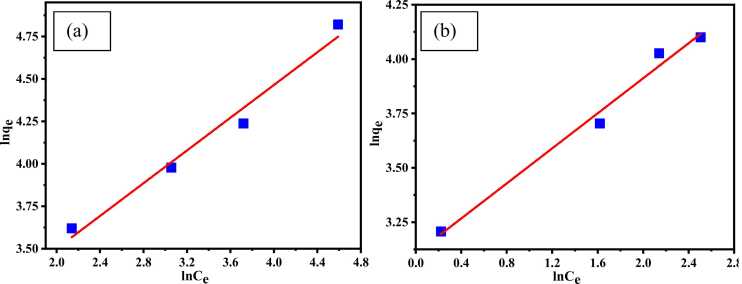
Freundlich isotherm for the removal of (a) Cr (III) and (b) AV54 dye with CA-PVA-GO composite.

Where, k_F_ = Freundlich isotherm constant (L/g), n = adsorption intensity, Ce = equilibrium concentration (mg/L), q_e_ = adsorption capacity at equilibrium (mg/g). k_F_ suggests multilayer adsorption capacity of adsorbent associated to bonding energy and 1/n represents adsorption intensity heterogeneity of the adsorbent sites [63]. k_F_ and n are designed from the graph *lnq*_*e*_ vs *lnC*_*e*_ where, n gives an assumption favorability of adsorption, to be either good adsorption if n=2–10, difficult if n= 1–2 and poor adsorption if n<1 [64], [65]. Fig. 12. showed the Freundlich isotherm for (a) Cr (III) and (b) AV54 dye removal by CA-PVA-GO composite.

The adsorption isotherm results are presented in Table 1. For Cr(Ⅲ) and AV54 dye adsorption, the Freundlich isotherm correlation coefficient values, R^2^ were 0.977 and 0.987, respectively, which represented the formation of multilayers on the adsorbent surface. The values of adsorption intensity, n = 2.074 and 2.480, represent a favourable adsorption process. The value of R^2^ for both isotherm and n followed the theoretical standard but was better fitted with dye adsorption since the batch experiment was carried out at a lower adsorbate concentration compared to Cr (III) adsorption [66].

**Table 1:** Adsorption isotherms parameters for Cr (III) and dye (AV54) removal by CA-PVA-GO composite.

**Model**	**Parameters**	**Cr(Ⅲ) removal**	**AV54 dye removal**
Langmuir	q_m_ (mg/g)	173.01	74.68
	b (L/mg)	0.0224	0.0134
	R^2^	0.898	0.975
	R_L_	0.182	0.999
Freundlich	K_f_	12.621	22.338
	n	2.074	2.480
	R^2^	0.977	0.987

### 3.4 Adsorption kinetics

It is essential to consider adsorption kinetics when evaluating the speed and effectiveness of the adsorption process. Two kinetic models were used in this study to explain the adsorption process. To characterize the kinetic process, Lagergren presented a pseudo-first-order (PFO) rate equation in 1998, and Ho and Mckay produced a pseudo-second-order (PSO) equation in 1995 [67].

#### 3.4.1 Pseudo-first-order kinetic

The linear appearance of PFO rate is as Eq. (6).

(6) $$\log\left(q_{e}-q_{t}\right)=\log q_{e}-\frac{k_{1}}{2.303}t$$

Here, q_e_= adsorption capacity at equilibrium (mg/g); q_t_ = adsorption capacity (mg/g) at time, t; k_1_= rate constant of Pseudo-First-Order adsorption reaction (L/min).

#### 3.4.2 Pseudo-second-order kinetic

The linear shape of PSO rate Eq. (7) is as follows:

(7) $$\frac{t}{q_{t}}=\frac{1}{k_{2}\;{q_{e}}^{2}\;}+\frac{1}{q_{e}}\;t$$

**Fig. 13:**
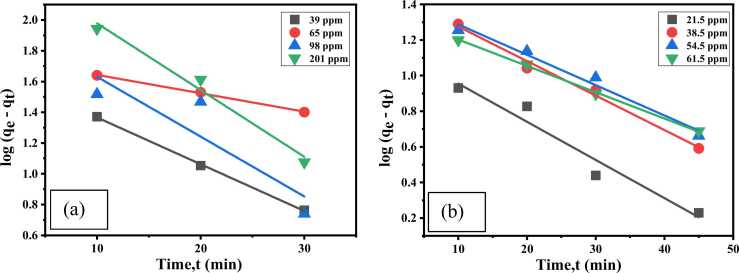
PFO kinetic for Cr(Ⅲ) and dye for (a) Cr (III) and (b) AV54 dye removal by CA-PVA-GO composite.

**Fig. 14:**
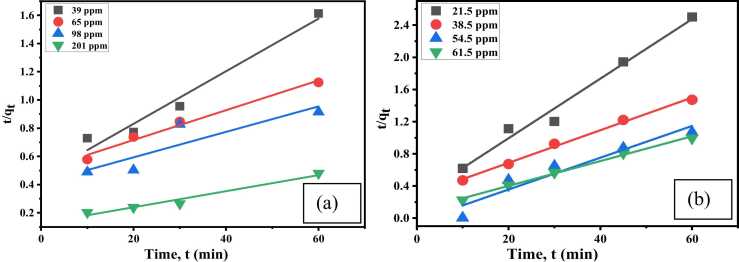
PSO kinetic for (a) Cr (III) and (b) AV54 dye removal by CA-PVA-GO composite.

Where, k_2_= the rate constant (g/mg min).Fig. 13, Fig. 14

#### 3.4.3 Explanation

The related adsorption kinetics parameters are shown in Table 2. For Cr(Ⅲ) adsorption, R^2^ values for the PFO were quite close to the highest correlation coefficient values (0.955<R^2^<0.999), and the theoretical q_e_ values agreed well with the experimental q_e_ values, which suggested the adsorption process followed the PFO model. However, for dye adsorption, the R^2^ values were 0.920–0.995, and theoretical q_e_ values agreed well with the experimental q_e_ values that represented the fulfilment of the PSO model [68], [69].

**Table 2:** Summary of adsorption kinetics for Cr (III) and dye (AV54) adsorption on CA-PVA-GO composite.

**Kinetics Model**		**PFO kinetic parameters**				**PSO kinetic parameters**		
Parameters	Conc. (ppm)	k_1_	R^2^	q_e_^a^ (mg/g)	q_e_^b^ (mg/g)	k_2_	R^2^	q_e_^b^ (mg/g)
For Cr(Ⅲ) removal	39.50	0.071	0.999	37.20	46.77	0.0007	0.968	53.76
	65.25	0.275	0.997	53.35	57.54	0.0022	0.984	95.24
	98.20	0.087	0.798	71.95	104.71	0.0875	0.789	107.53
	201.75	0.099	0.981	125.00	257.04	0.0990	0.967	175.43
For dye removal	21.50	0.049	0.949	24.70	14.79	0.0054	0.981	27.03
	38.50	0.045	0.988	40.79	29.42	0.0018	0.995	49.31
	54.50	0.039	0.979	56.10	28.70	0.0099	0.920	50.76
	61.75	0.034	0.998	60.37	22.40	0.0025	0.995	64.85

q_e_^a^ is the experimental adsorption capacity and q_e_^b^ is the theoretical adsorption capacity.

### 3.5 Thermodynamics study

In this research the changes in Gibbs free energy for adsorption on adsorbents at various temperature were calculated by following (8), (9) [70].

(8) $$∆G=-\mathrm{RT}\ln k_{d}$$

Where, k_d_ = distribution constant for the equilibrium sorption, R = universal gas constant (8.314 J mol^−1^K^−1^) and T = absolute temperature (K). k_d_ was deliberated by the following equation.

(9) $$k_{d}=\frac{q_{e}}{C_{e}}\;$$

The average standard enthalpy changes ΔH and entropy change ΔS for the adsorption was designed by van’t Hoff Eq. (10).

(10) $$\ln k_{d}=\frac{∆S}{R}-\frac{∆H}{\mathrm{RT}}$$

lnk_d_ versus1/T was plotted to calculate ΔH and ΔS. Parameters ∆H and ∆S represents the entropy change can be calculated from thermodynamics Eq. (10) using the slope and intercepts. The theoretical data indicates that the reaction is unfavorable at high temperatures. Negative enthalpy defines the exothermic characteristic of the reaction. With the increasing temperature, Gibbs free energy is decreased, gradually ensuring the spontaneous character of the adsorption, and at a higher temperature, the reaction is unfavorable [51].

To investigate the effect of different temperatures, 0.82 g/L CA-PVA-GO was added to 20 mL of (45 ppm) Cr_2_(SO4)_3_.6 H_2_O solution at pH 5.0 and 20 mL of (45 ppm) AV45 dye solution at pH 3.0 for each experiment. The batch experiments were then shaken in an orbital shaker for 60 minutes at 298, 308, 318, and 328 K.

**Fig. 15:**
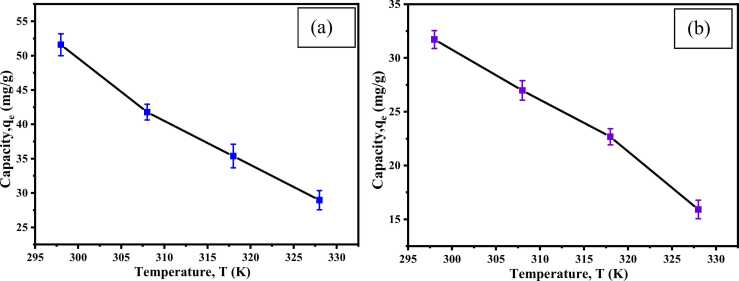
Effect of temperature on (a) Cr (III) and (b) AV54 dye removal by CA-PVA-GO composite.

**Fig. 16:**
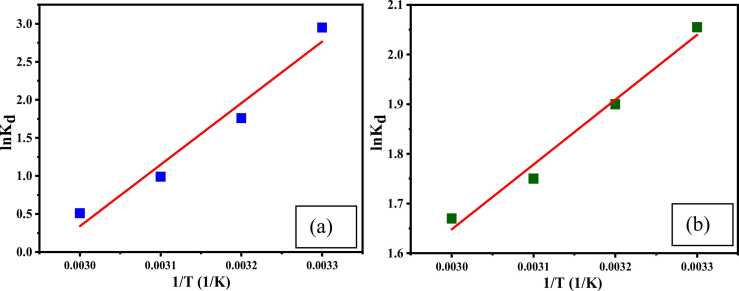
Effect of temperature for Cr(Ⅲ) and dye (a), (b); and Vant Hoff՚s equation for Cr(Ⅲ) and dye.

Maximum adsorption capacities were 51.59 ± 1.59 and 31.71 ± 0.83 mg/g (Fig. 15, Fig. 16 (a) and (b)) at 298 K for Cr (III) and AV54 dye, respectively, which decreased to 28.96 ± 1.39 and 15.91 ± 0.86 mg/g at 328 K. When the temperature was raised, the kinetic energy increased and released the adsorbate from CA-PVA-GO [71].

From Fig. 15 (a), (b), and Table 3, the values of ΔG for adsorption of Cr (III) and AV54 dye on CA-PVA-GO at temperatures of 298, 308, 318, and 328 K were found to be −7.433, −4.508, −2.626, −1.311 kJ/mol, and −5.178, −4.861, −4.628, and −4.555 kJ/mol, respectively. The negative ΔG suggests that the adsorption of Cr (III) and dye on CA-PVA-GO was spontaneous, and the negative ΔH implies the exothermic nature of the process. Moreover, during the adsorption, the negative ΔS signifies the reduction in randomness at the solid/solute interface.

**Table 3:** Parameters for Van’t Hoff՚s equation for Cr (III) and dye (AV54) removal on CA-PVA-GO composite.

**Parameters**	**∆G (kJ/mol)**				**R**^**2**^	**∆H (kJ/mol)**	**∆S (kJ/mol)**
	**298 K**	**308 K**	**318 K**	**328 K**			
**Cr(Ⅲ) removal**	-7.433	-4.508	-2.626	-1.311	0.963	-67.257	-0.198
**Dye removal**	-5.178	-4.867	-4.628	-4.555	0.981	-10.852	-0.019

### 3.6 Regeneration

To find out the possibility of reusing CA-PVA-GO as an adsorbent, Cr (III) and AV 54 dye-loaded CA-PVA-GO were regenerated using a 1.0 M HCl and NaOH solution three times. To investigate the capacity of regenerated CA-PVA-GO, further adsorption processes were studied at optimum conditions. For regeneration of Cr (III)-loaded CA-PVA-GO, 0.82 g/L CA-PVA-GO was added to 20 mL of a 50 ppm Cr_2_(SO_4_)3.6 H_2_O solution at 5.0 pH. In the case of dye-loaded CA-PVA-GO regeneration, all optimum conditions were the same except pH, which was 3.0.

Table 4 presents the findings of regeneration studies, which revealed that the adsorption capacity of regenerated adsorbents from Cr (III) and AV 54 dye-loaded CA-PVA-GO gradually decreased from 53.75 to 24.18 mg/g and 42.93–19.83 mg/g, respectively. The values signified that regenerated CA-PVA-GO would be reused for Cr (III) and AV 54 dye removal from the wastewater.

**Table 4:** Adsorption capacity of regenerated CA-PVA-GO.

**Regenerated** **CA-PVA-GO**	**Adsorption capacity (mg/g)**	
	**Cr (III) loaded**	**AV 54 dye loaded**
Fresh CA-PVA-GO	53.75	42.93
1st Recycle	47.16	33.19
2nd Recycle	29.73	25.51
3rd Recycle	24.18	19.83

### 3.7 Application of CA-PVA-GO on real sample (chrome tanning and dyeing effluent)

After justifying the capabilities of CA-PVA-GO, the performance of the removal adsorbate from the real sample was analyzed. To investigate the adsorption of chromium and dye from concentrated chrome tanning and dyeing effluent, 5 g of CA-PVA-GO was added to 500 mL of chrome tanning effluent at optimum pH 5.0 and 2 g of CA-PVA-GO was added to 500 mL dyeing effluent at optimum pH 3.0, separately and shaken at room temperature for 60 min. The concentration of chromium before and after adsorption was analyzed by ICP-MS, and the dye concentration was examined by UV–visible Spectroscopy. However, other water quality parameters such as pH, TDS, EC, BOD5, and COD were also tested, and the values were represented in Table 5, Table 6.

**Table 5:** Quality parameters of chrome tanning and dyeing effluents before and after adsorption.

	**Chrome tanning effluents**			**Dyeing effluents**		
**Parameters**	**Before (Cr) adsorption**	**After (Cr) adsorption**	**% of removal**	**Before (Dye) adsorption**	**After (Dye) adsorption**	**% of removal**
Cr total (ppm)	2874.83	1942.36	53.18	-	-	-
Dye (ppm)	-	-	-	232.16	14.74	93.91
q_m_ (mg/g)	-	93.24	-	-	54.36	-
pH	4.6	5.1	-	3.0	3.4	-
TDS (ppm)	7723	3153	59.17	2652	174	93.44
EC (µS/cm)	4507	1892	58.02	1458	131	91.02
BOD_5_ (ppm)	1273	497	60.95	723	109	84.92
COD (ppm)	4876	2091	57.11	2547	446	82.49

**Table 6:** Adsorption efficiency of different composites.

**Composite**	**Adsorbate**	**Adsorption capacity**	**References**
GO/PVA/SA	MB dye	45.56 mg/g	[73]
Mag-Ben/CCS/Alg	Cu (II)	56.79	[74]
SA/PVA/GO	Cu (II) and U (VI)	247.16 and 403.78 mg/g	[75]
GO/CMC-Alg	Cu (II)	73.27 mg/g	[76]
(PVA), (CMC) and ZSM-5 zeolite	MB dye	7.83 mg/g	[77]
PVA/CMC/ GO and bentonite	MB dye	172.14 mg/g	[78]
Zeolite/Fe2O3 nanocomposite	BV 16 dye	175.43 mg/g	[49]
PVA/Alg/CS	Pb (II) and Cr (VI)	139.40 and 86.10 mg/g	[79]
CA-PVA-GO	Cr (III) and AV54 dye	173.01 and 74.68 mg/g	This study

The obtained results revealed that after adsorption, chromium and dye removal% were 53.18 and 93.91%, respectively; however, the adsorption capacities were 93.24 and 56.86 mg/g, respectively, which were much lower compared to the theoretical adsorption capacity (173.01 mg/g for Cr and 74.68 mg/g for dye adsorption). This happened due to the existence of aggressive ions and other matters that were used in leather manufacturing, which might decrease the capacity of the CA-PVA-GO [72]. However, the water quality parameters like pH, TDS, EC, BOD5, and COD of the analyzed Cr-tanning and dyeing effluents were significantly improved after adsorption with CA-PVA-GO.

## 4 Conclusions

This study utilized biodegradable, water-soluble, and environment-friendly PVA, sodium alginate, CaCl_2_, and GO to produce the adsorbent CA-PVA-GO, which was utilized for the adsorption of Cr (III) and AV54 dye. A different batch experiment was performed, and the optimized pH, adsorbent dosage, and contact time were revealed to be 5.0, 0.8 g/L, 45 min for Cr (III), and 3.0, 0.8 g/L, 60 min for dye adsorption, respectively. At optimum pH and dosage, Cr (III) and AV54 dye removal percentages were found to be 35.35 ± 1.43% and 84.63 ± 2.54%, respectively. The impact of initial concentration and temperature was also investigated. The findings revealed that the adsorption capacity of CA-PVA-GO decreased with higher adsorbent dosages and increased with increasing initial adsorbate concentrations. The maximum adsorption capacity for Cr (III) and AV54 dye removal was 173.01 and 74.68 mg/g, respectively. The pseudo-first-order kinetic model was better fitted with experimental data for Cr (III) adsorption (R^2^ = 0.798–0.999), but the pseudo-second-order model was followed for AV54 dye adsorption (R^2^ = 0.92–0.995). Experimental results of the thermodynamic study indicate that Cr (III) and the dye adsorption process were exothermic and spontaneous due to the negative values of ΔG and ΔH. Real sample analysis revealed that the adsorption capacity and removal percentage of CA-PVA-GO for chromium and AV54 dye adsorption from chrome tanning and dyeing effluents were 93.24 and 54.36 mg/g, and 53.18 and 93.91%, respectively. The results also stated that CA-PVA-GO has the proficiency to remove (> 50%) various pollution parameters, including TDS, EC, BOD_5_, and COD. Though the CA-PVA-GO composite showed good potentiality and advantages for the removal of Cr (III) and AV54 dye, some limitations, such as limited adsorption capacity and percentage of removal, the performance of the regenerated adsorbent, and the pH of the effluent, are yet to be overcome. However, the environmental friendliness and biodegradability of CA-PVA-GO composite could be an appreciated approach for tannery wastewater treatment.

## Declaration of Competing Interest

The authors declare that they have no competing financial interests or personal relationships that could have appeared to influence the work reported in this paper.

## Data Availability

No data was used for the research described in the article.
